# Comparing Temporary Immobilization Using Cast and External Fixator in Unimalleolar Ankle Fracture Dislocations: A Retrospective Case Series

**DOI:** 10.3390/jcm12030748

**Published:** 2023-01-17

**Authors:** Eric Mandelka, Bernhard A. Wikanardi, Nils Beisemann, Paul A. Gruetzner, Jochen Franke, Sven Y. Vetter, Maxim Privalov

**Affiliations:** 1 Research Group Medical Imaging and Navigation in Trauma and Orthopedic Surgery (MINTOS), BG Klinik Ludwigshafen, Ludwig-Guttmann-Strasse 13, 67071 Ludwigshafen, Germany; 2 Medical Faculty of Heidelberg, Ruprecht-Karls-Universität Heidelberg, Im Neuenheimer Feld 672, 69120 Heidelberg, Germany

**Keywords:** ankle fracture dislocation, primary management, loss of reduction, secondary dislocation, cast, external fixator, complications

## Abstract

Studies have reported a high percentage of ankle fracture dislocations with secondary loss of reduction during primary treatment with a splint or cast. This study aimed to assess the rate of secondary loss of reduction in unimalleolar ankle fracture dislocations treated primarily with a cast or external fixator, identify the potential influence of fracture morphology, and investigate the potential implications. Unimalleolar ankle fracture dislocations with and without posterior malleolar fracture between 2011 and 2020 were included. Patients were categorized into two groups, depending on the method of temporary treatment. Fracture morphology, time to definitive surgery, and soft-tissue complications were compared. Of 102 patients, loss of reduction tended to occur more often in the cast group (17.3%) than in the external fixator group (6.0%). The presence of a posterior malleolar fracture did not have a significant influence on loss of reduction in cast immobilization; however, the fragment proved to be significantly bigger in cases with loss of reduction. No statistically significant differences in soft tissue complications or time to definitive surgery were found. Surgeons should consider the application of interval external fixation in the primary treatment of unimalleolar ankle fracture dislocations with additional posterior malleolar fractures.

## 1. Introduction

With an incidence of approximately 100 per 100,000 person–years, ankle fractures are among the most common fractures in adults [1,2]. In most cases, the definitive treatment for ankle fractures is open reduction and internal fixation (ORIF). This is especially the case in fractures with primary dislocation of the upper ankle joint resulting in loss of congruence of the tibiotalar joint surfaces; however, posttraumatic soft tissue swelling prevents definitive therapy from being performed immediately [3]. In such patients, obtaining early reduction and temporary immobilization of the fracture until definitive surgical treatment can be performed is key in primary fracture management [4]. This is often accomplished by the application of a lower leg splint or a bivalved cast, and subsequent discharge of the patient, with planned re-presentation for surgery after soft-tissue swelling decrease.

However, this form of temporary fracture management may not be appropriate in every case as ankle fractures are often unstable in multiple planes [5]. Accordingly, secondary dislocation after closed reduction and splint immobilization of ankle fractures has been described by Matson et al., who reported loss of reduction (LOR) in 43% of patients after primarily successful closed reductions [6].

LOR not only leads to more pain but potentially increases the risk for complications due to soft-tissue contracture as well as osteochondral lesions. In addition, the reduced decrease in swelling prolongs the time to definitive surgery [7]. As a result, especially in primarily dislocated ankle fractures, alternatives to temporary splint immobilization have been investigated [8]. More recent studies have examined LOR rates in patients with ankle fracture dislocation and temporary treatment with a lower-leg plaster splint or external fixation (ExFix). Both Buyukkuscu et al. and Wawrose et al. reported significantly higher LOR rates, 25% and 50%, respectively, in patients temporarily treated with a lower leg splint compared with interval ExFix [7,9].

While Wawrose et al. did not analyze the effect of fracture morphology, Buyukkuscu et al. found the rate of loss of ankle joint reduction to be significantly higher in the presence of a fracture of the posterior malleolus. However, the authors did not differentiate between unimalleolar and bimalleolar ankle fractures. This could be of importance in assessing the individual risk of secondary dislocation and the occurrence of potential complications as well as in potentially revising the appropriate treatment algorithms for different fracture morphologies. Therefore, the purpose of this study was to compare the rate of LOR in unimalleolar ankle fracture dislocations for both methods of primary fracture immobilization. Furthermore, we aimed to investigate the influence of fracture morphology on the LOR rate, as well as the rate of pre and postoperative complications, for both groups.

It was hypothesized that immobilization in a cast leads to a significantly higher LOR rate compared with interval ExFix, and that this rate is higher in the presence of an additional fracture of the posterior malleolus.

## 2. Materials and Methods

In this retrospective case series, after ethics approval, a list of all patients treated at our level 1 trauma center between January 2011 and December 2020 with the ICD diagnosis codes S82.4, S82.5, S82.6, S82.7, S82.81, S82.82, S82.88, and S82.9 was reviewed in detail to identify patients with primarily dislocated unimalleolar ankle fractures. Ankle fracture dislocation was defined as a loss of congruity of the upper ankle joint space, depicted by an enlargement of the medial clear space of and/or anteroposterior (subluxation) of the talus relative to the tibia of ≥5 mm anteroposterior (sub)luxation of the talus relative to the tibia, based on the first radiographs that were performed. If these criteria were met, immediate closed reduction of the ankle joint was performed under radiographic control in our institution to restore ankle joint congruity, thereby avoiding secondary damage to the joint and the surrounding soft tissue. Cases without joint dislocation, as well as patients with open fractures, non-operative definitive fracture management, or ORIF performed within 48 h after admission or in an external institution, were excluded. Additionally, patients were excluded when existing medical image data did not include radiographs proving initial ankle fracture dislocation and/or correct reduction after application of cast or ExFix.

The remaining cases were categorized into two groups, depending on the method of initial fracture management. LOR was chosen as the primary study endpoint. Subgroups of the two treatment groups were formed with regard to the occurrence of the primary endpoint. All medical records, including X-ray, intraoperative 3D imaging, CT, and MRI imaging, were reviewed for the patients included in the study. The presence and size of a posterior malleolus fracture (fragment size to joint surface ratio) and time duration between initial treatment and definitive ORIF were investigated. The medical documentation was reviewed for pre and postoperative occurrence of soft-tissue complications, such as skin necrosis, infection, wound dehiscence, prolonged wound secretion, acute compartment syndrome, or revision surgery, which were then compared between the two groups.

In the first group, closed reduction and ExFix was performed under fluoroscopic control in the operating room by a board-certified orthopedic and trauma surgeon and an orthopedic resident. ExFix was applied using two pins in the tibia shaft, one threaded Steinmann pin in the calcaneus, and one pin each in the base of the first and fifth metatarsal bones [5]. Afterwards, all patients were admitted for soft-tissue control, remaining hospitalized until ORIF was performed.

**Figure 1:**
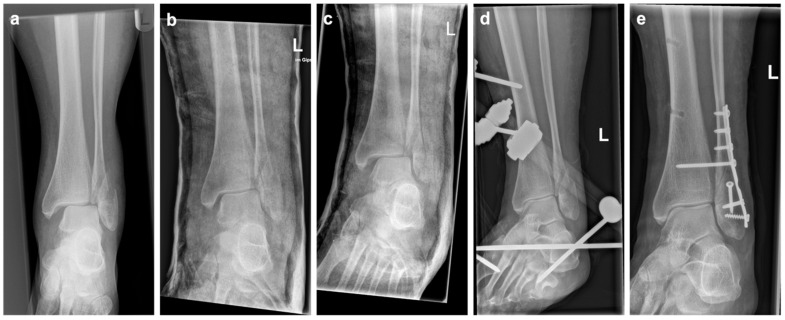
Example of a unimalleolar ankle fracture dislocation, with radiographs taken prereduction (**a**), postreduction with improved joint position in the cast (**b**), and after loss of reduction in the cast 3 days later (**c**). Subsequently, ExFix was applied (**d**) before definitive ORIF was performed (**e**).

In the second group, a plaster cast was applied for immediate fracture immobilization after fluoroscopically controlled closed reduction in the emergency department. As soon as the plaster cast had dried, radiographic imaging was performed to assess adequate reduction, with a medial clear space of <5 mm and uniform width of the tibiotalar and tibiofibular joint space (Figure 1) [6]. Directly afterwards, medial and lateral cuts were applied to the cast, in accordance with AO/OTA principles. After applying the cuts, the cast was held in place by elastic bandages to allow for posttraumatic soft-tissue swelling. All patients were admitted as inpatients for elevation and soft-tissue control.

In case of (persistent) dislocation in postreduction radiographs, these events were not considered secondary LOR because there was no radiographic proof that, after cast application, adequate reduction of the ankle joint had been restored. In all these cases, immediate treatment with ExFix was performed. Consequently, these patients were included in the ExFix group.

Standard radiographic imaging was performed two days after application of a cast or ExFix to check for persistence of ankle joint reduction. Standard radiographic imaging was performed two days after application of a cast or ExFix to check for persistence of ankle joint reduction. Additionally, radiographic imaging was performed at any point in case of clinical suspicion of LOR. The timing of definitive ORIF was based on reductions in swelling and soft-tissue condition.

Sample size calculation was performed using G*Power 3.1. Based on the results of Wawrose et al., Buyukkuscu et al., and Gerlach et al., a total sample size of *n* = 88 was calculated (two-tailed Fisher’s exact test; power 0.9; alpha error probability 0.05).

Statistical analysis was performed using SPSS (IBM SPSS version 27.0.1.0, Chicago, IL, USA). Figures were created using Prism 8 (Graphpad Software, Version 8.2.1, San Diego, CA, USA). The Shapiro–Wilk test was used to check for normal distribution of data. Descriptive statistics were reported as means with standard deviation.

Age, posterior malleolus fragment size, and time to ORIF in the treatment groups were compared using the Mann–Whitney U test. Fisher’s exact test was used to investigate the contingency of categorical data, as in gender and AO/OTA classification, as well as associations between probability of loss of reduction, posterior malleolus fracture, and complications between groups and subgroups. Differences in time to ORIF in the subgroups were analyzed using the Kruskal–Wallis test, with Dunn’s post hoc correction for multiple comparisons. The ExFix with LOR subgroup was excluded from this analysis for statistical reasons (small sample size). For all statistical tests, significance was set at *p* < 0.05.

## 3. Results

In total, 102 patients with dislocated unimalleolar ankle fractures were included in the study. The demographic data of the patients, as well as fracture classification according to the AO/OTA classification, showed no significant differences between the groups (Table 1). In 52 patients, a plaster cast was applied for temporary immobilization, while 50 cases were included in the ExFix group. For the latter group, ExFix was used as the first-choice method in 32 cases (64%). In the other 18 cases (36%), ExFix application was performed because of postreduction and cast application radiographs showing inadequate reduction.

### 3.1. Secondary LOR

**Figure 2:**
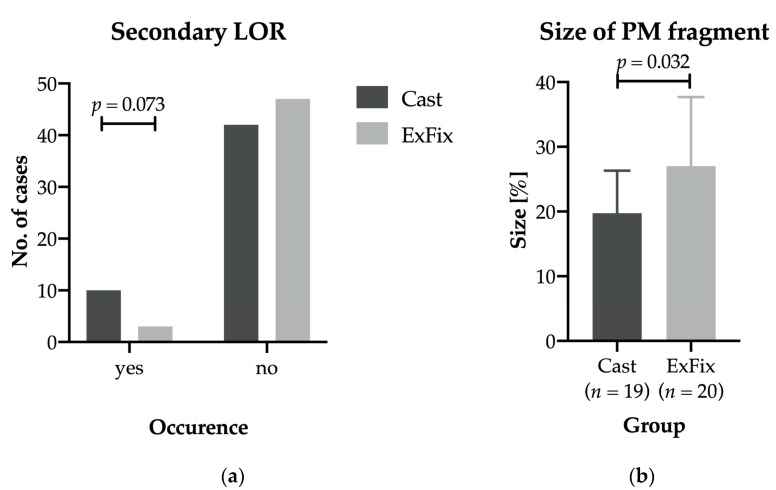
Comparison of occurrence of secondary loss of reduction (**a**) and mean size of the posterior malleolar fragment (**b**) for both groups. PM: posterior malleolar.

LOR occurred in nine patients in the cast group and three patients in the ExFix group. Therefore, LOR tended to occur more often after application of the cast than after application of interval ExFix; however, this difference did not reach statistical significance (Figure 2a). All three patients suffering LOR in the ExFix group subsequently underwent revision of ExFix.

### 3.2. Influence of a Posterior Malleolar Fracture

Comparison of the two treatment groups regarding the presence of an additional posterior malleolar fracture showed no significant difference (*p* = 0.839). However, the mean size ratio of the posterior malleolar fracture fragment compared with the total joint surface was significantly smaller in the cast group compared with the ExFix group (19.8 ± 6.5% vs. 27.0 ± 10.7%, Figure 2b). The influence of the posterior malleolar fracture and the fragment size ratio on the probability of LOR was further investigated. No significant difference was found in the treatment groups for the probability of LOR when a posterior malleolar fracture was present (*p* = 0.293 for the cast group and *p* > 0.99 for the ExFix group). However, the fragment size ratio in the cast group was significantly higher in cases with LOR than in cases without LOR (*p* = 0.023, Table 2). In the ExFix group, the influence of the posterior fragment size ratio on LOR was not determined for statistical reasons (LOR *n* = 1).

### 3.3. Time to Definitive ORIF

**Figure 3:**
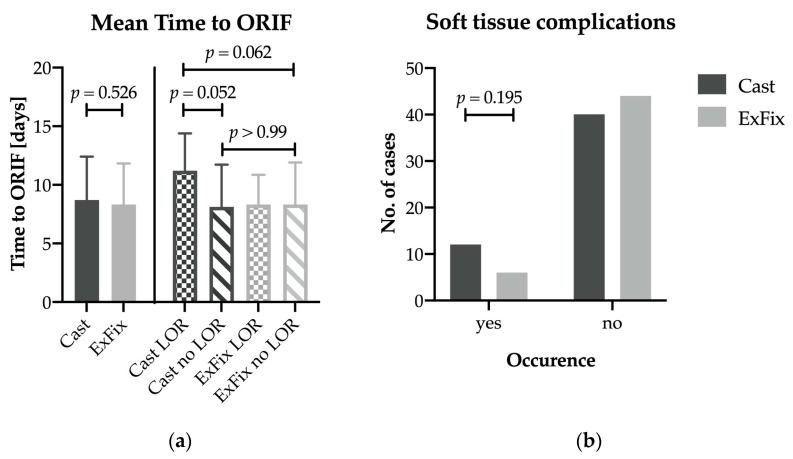
(**a**) Mean time to ORIF for both groups and subgroups depending on secondary LOR. The subgroup, ExFix with LOR, was not considered in statistical testing because of the limited group size of *n* = 3. (**b**) Comparison of occurrence of soft-tissue complications in both groups. ORIF: open reduction and internal fixation.

The mean time to definitive surgery was 8.7 ± 3.7 days for the cast group and 8.3 ± 3.5 days for the ExFix group, showing no significant difference (*p* = 0.526). Similarly, there was no significant difference in the post hoc comparison of the subgroups, cast with LOR (11.2 ± 3.2 days), cast without LOR (8.1 ± 3.6 days), and ExFix without LOR (8.3 ± 3.6 days), when adjusted for multiple comparisons (Figure 3a).

### 3.4. Complications

Soft-tissue complications were observed in 12 cases (23.1%), with a total of 20 complications in the cast group in 6 cases (12.0%, *p* = 0.195) and a total of 8 complications in the ExFix group (Figure 3b, Table 3). Revision surgery had to be performed in 8 cases in the cast group, while 3 patients in the ExFix group had to undergo repeated surgery.

## 4. Discussion

Compared with the extensive number of publications on ankle fractures in general, ankle fracture dislocations remain a rather understudied topic in the literature [10]. The recently published systematic review by Cao et al. included only 15 studies on ankle dislocation fractures, with only two examining the initial management of this injury [11]. The studies conducted by Wawrose et al. and Buyukkuscu et al. have reported secondary LOR rates of 25% and 50%, respectively, for ankle fracture dislocations with temporizing splint immobilization, which is significantly higher than the LOR rates for the respective ExFix groups [7,9]. A third study performed by Gerlach et al., however, found no significant difference in secondary LOR when using a cast instead of a splint compared with temporary ExFix [12].

The influence of a posterior malleolar fracture was investigated by both Buyukkuscu et al. and Gerlach et al.; however, these studies either did not distinguish between unimalleolar and bimalleolar fractures or did not include unimalleolar fractures at all. To the best of the authors’ knowledge, there are no studies in the literature reporting on the specific LOR rates for unimalleolar ankle fracture dislocations.

The most important finding of our study is that although the LOR rate was higher in the cast group, this difference was not statistically significant, as was reported by recently published studies. There are several reasons that could explain why our study hypothesis could not be confirmed.

First, the difference with regard to the LOR rate could be due to the fact that the literature lacks a uniform definition of the term “ankle fracture dislocation.” Inclusion criteria range from “more than 33% or 50% subluxation of the talus” to unspecified criteria such as “loss of opposition of articular surfaces” or “total loss of articular congruity” [4,7,8,9,10,13,14,15]. Notwithstanding, in our study, we decided to apply a far lower cut-off value for subluxation because only a small number of patients are admitted to the emergency room at our institution with persisting severe dislocation of the ankle. Therefore, fractured ankles may not appear as dislocated in the first radiographs that are taken; however, the severity of the initial injury, as well as the resulting degree of instability, may be the same. This is confirmed by our results, proving that LOR in cast immobilization is still a common problem, even in fractures with a small degree of subluxation. Admittedly, patients were excluded when fractures were anatomically reduced initially and when reduction was maintained throughout the initial radiographic assessment. However, we believe that this affected only few patients.

Second, contrary to the results in the literature, the present study focused solely on the LOR rate of unimalleolar fractures, which may not be as prone to secondary dislocation as bimalleolar fractures given that the direction of instability is limited by the intact second malleolus. As existing studies did not differentiate between uni and bimalleolar ankle fracture dislocations, this restriction was made in order to obtain data that allow for the specific analysis of primary fracture management.

Third, the current study differed from those conducted by Wawrose et al. and Buyukkuscu et al. in some respects regarding the procedure. While lower-leg plaster splints were used in these studies, bivalved lower-leg plaster casts are applied at our institution by default. In addition to providing space for potentially increasing soft-tissue swelling, this technique ensures greater stability than a conventional splint. This was shown in a study by Baker et al. on bivalved fiberglass casts compared with conventional plaster splints, for which a combination of posterior and sugar-tong/stirrup splints was used [16].

Despite the preselection detailed in the Section 2, almost every fifth fracture immobilized in a cast experienced secondary dislocation. With interval ExFix, LOR occurred in only 6.0% of patients. This is in accordance with results presented by Shah et al. and Buyukkuscu et al., who reported LOR of 0.0% and 4.0%, respectively, in the ExFix groups [5,7]. Whether LOR was a result of insufficient application of the ExFix or patient incompliance could not be determined retrospectively in our study collective. In all cases, revision of the ExFix was performed in the operating room, with repeated joint reduction.

Following the application of ExFix or the cast, all patients were admitted and remained as inpatients for elevation and soft-tissue control until ORIF was performed. In contrast, in the study by Buyukkuscu et al., patients from both groups were discharged between temporary and definitive treatment, with outpatient follow-up advised. This may have reduced patient compliance regarding, for example, weight-bearing or splint removal [7]. These differences in protocol may have had an influence on the LOR rate.

While patient outcomes were not part of our retrospective data analysis, several authors have reported the outcomes of ankle fracture dislocations to be statistically poorer compared with non-dislocated ankle fractures as a consequence of the more severe injury to the soft tissue surrounding both the bone and ankle [8,10,14,17]. This becomes evident when comparing complication rates with non-dislocated ankle fractures. In 1991, Carragee et al. documented a wound complication rate of 44% following dislocation, which corresponded to a threefold increased risk compared with patients without dislocation [18]. Since then, different studies have compared complication rates and identified trends; however, no statistically significant differences have been reported [10,14,19].

To date, no studies have been published investigating whether long-term outcomes depend solely on definitive ORIF or whether this is influenced by primary fracture management, including the quality of the initial reduction. Nevertheless, as stated above, we applied strict criteria for both the initial reduction and secondary loss of reduction to avoid secondary damage to the joint and soft tissue.

In line with other studies, we found a tendency for the overall rate of soft-tissue complications to be higher in the cast group compared with the ExFix group, despite the fact that the use of ExFix is associated with additional risk of pin infection and iatrogenic fractures [20]. As the rate of LOR was also significantly higher after interval splinting, a correlation could be implied. However, data have not been furnished by other authors to support this assumption thus far, nor could we establish any correlation between LOR and subsequent complications from our data [7,9].

In contrast, the association between a fracture of the posterior malleolus and not only initial dislocation, but also secondary LOR during the course of splinting treatment, seems to be clearly documented [7,14,17]. More specifically, Matson et al. reported that 65% of splinted fractures experienced LOR when the fragment size ratio was >0.1 [6]. In the current study, in general, no significantly higher LOR rate was found in the presence of a posterior malleolus fracture; yet, in the cast group, the fragment size ratio was significantly higher for cases with LOR.

For both groups and subgroups, no significant differences with regard to the time to definitive treatment were found in our study. In a systematic review performed by Cao et al., the pooled mean time from injury to surgery was 7.2 days; however, the authors did not distinguish between different methods of primary immobilization [11].

Various authors have concluded that ExFix may be the more appropriate interval treatment for ankle fracture dislocations because it significantly reduces the frequent phenomenon of secondary LOR observed in interval splinting [7,9]. Although our inclusion criteria differed considerably from the aforementioned studies and our protocol resulted in only a preselected collective receiving interval treatment with the cast under controlled conditions, secondary dislocations tended to occur more frequently compared with the ExFix group. In addition, the rate of soft-tissue complications tended to be higher in the cast group. Accordingly, in cases of pre-existing soft-tissue complications or doubts concerning the stability of the fracture in cast immobilization, the application of ExFix should not be limited to bimalleolar fractures but performed in unimalleolar ankle fracture dislocations as well. Moreover, interval treatment with a bivalved plaster cast may be an adequate alternative for the temporary treatment of preselected ankle fracture dislocations and should be compared with conventional splints in further studies.

The present study has some limitations that should be noted. First, because of its retrospective nature and the limited information available, the investigation is prone to bias. Consequently, to prevent selection bias due to false documentation, we included only cases in which pre and postreduction radiographs, as well as radiographs proving secondary loss of reduction, were available for review. Furthermore, all radiographic evaluations were performed systematically by a single investigator and then confirmed by the first author. In addition, our results are biased by the fact that the decision on primary treatment was made by dozens of different treating surgeons over the inclusion period of 10 years. Consequently, our results may be more transferable to other institutions as they are the result of an individual decision-making process.

Finally, our investigation does not include patient follow-up after definitive ORIF, which would indicate clinical outcomes, because the investigation focused on loss of reduction during interval treatment. However, these results would probably be biased anyway because of the individual quality of open reduction and internal fixation performed. Further studies with larger sample sizes are needed to assess both the risk factors as well as the consequences of secondary loss of reduction in terms of patient outcomes.

## 5. Conclusions

In conclusion, we found a clear trend showing higher risk of secondary loss of reduction in temporary treatment with a bivalved plaster cast compared with interval external fixation for unimalleolar ankle fracture dislocations. The presence of a posterior malleolar fracture did not have a significant influence on the loss of reduction rate in cast immobilization; however, the posterior malleolar fragment proved to be significantly bigger in cases with secondary loss of reduction. No significant differences were found between the groups regarding time to definitive ORIF and the rate of soft-tissue complications.

Surgeons should consider the application of interval external fixation in the primary treatment of unimalleolar ankle fracture dislocations with additional posterior malleolar fractures.

## Figures and Tables

**Table 1:** Demographics and injury characteristics. AO/OTA: Arbeitsgemeinschaft für Osteosynthesefragen/Orthopaedic Trauma Association.

	Cast (*n* = 52)		ExFix (*n* = 50)		*p*
Age [years]	43.9 ± 15.3		50.2 ± 18.1		0.080 *
Gender					
Male [%]	34	(65.4)	33	(66.0)	>0.99 ^+^
Female [%]	18	(34.6)	17	(34.0)	
AO/OTA classification					
44B1 [*n* (%)]	9	(17.3)	5	(10.0)	0.415 ^+^ (for B/C)
44B2 [*n* (%)]	18	(34.6)	19	(38.0)	
44B3 [*n* (%)]	4	(7.7)	10	(20.0)	
44C1 [*n* (%)]	14	(26.9)	12	(24.0)	
44C2 [*n* (%)]	6	(11.5)	4	(8.0)	
44C3 [*n* (%)]	1	(1.9)	0	(0.0)	
Posterior malleolus fracture					
yes [*n* (%)]	19	(36.5)	20	(40.0)	0.839 ^+^
no [*n* (%)]	33	(63.5)	30	(60.0)	

* Mann–Whitney test. ^+^ Fisher’s exact test.

**Table 2:** Relationship between fractures of the posterior malleolus and loss of reduction (LOR) in both treatment groups.

	No LOR		LOR		*p*
Cast					
No. of patients [*n*]	42	(100)	10	(100)	0.073 ^+^
Posterior malleolar fracture [*n* (%)]	17	(40)	2	(20)	0.293 ^+^
Posterior fragment size ratio [%]	18.4 ± 5.2		31.2 ± 6.6		0.023 ^+^
ExFix					
No. of patients [*n*]	47	(100)	3	(100)	0.073 ^+^
Posterior malleolar fracture [*n* (%)]	19	(40)	1	(33)	>0.99 ^+^
Posterior fragment size ratio [%]	26.6 ± 10.8		35.0 ± 0.0		– *

Statistical testing not possible because of small sample size. * Mann–Whitney test. ^+^ Fisher’s exact test.

**Table 3:** Overview of soft-tissue complications in both groups.

	Cast (*n* = 52)		ExFix (*n* = 50)		*p*
Cases with soft-tissue complications [*n* (%)]	12	(23.1)	6	(12.0)	0.195 ^+^
Total number of soft-tissue complications [*n* (%)]	20	(38.5)	8	(16.0)	0.015 ^+^
Skin necrosis [*n* (%)]	0	(0.0)	0	(0.0)	0.999 ^+^
Infection [*n* (%)]	2	(3.8)	1	(2.0)	0.999 ^+^
Wound dehiscence [*n* (%)]	4	(7.7)	1	(2.0)	0.363 ^+^
Prolonged wound secretion [*n* (%)]	5	(9.6)	3	(6.0)	0.716 ^+^
Acute compartment syndrome [*n* (%)]	1	(1.9)	0	(0.0)	0.999 ^+^
Revision surgery [*n* (%)]	8	(15.3)	3	(6.0)	0.201 ^+^

^+^ Fisher’s exact test.

## Data Availability

All data and statistics are available upon request from the corresponding author.
